# Surgical Problems in Intra-Pelvic and Abdominal Diseases

**Published:** 1894-07

**Authors:** A. H. Cordier

**Affiliations:** Kansas City, Mo.


### SURGICAL PROBLEMS IN INTRA-PELVIC AND ABDOMINAL DISEASES.

By A. H. CORDIER, M. D., Kansas City, Mo.

[From the *Kansas City Medical Recorder*, March, 1894.]

Some time ago, while doing special work in a large Eastern city, I had many opportunities to see the work of the various operators, and it was a noticeable fact that, while one operator would have a mixture of the worst neglected and complicated cases imaginable, including old adherent and caseous Fallopian tubes and ovaries in emaciated and septic patients, another operator's cases would be confined to the removal of cystomas and plastic vaginal and cervical cases. This led me to inquire of one of the operators why he did not have so many "pus cases" as some of his *confrères*. He replied that he believed that "the woods are *not* full of them," as some would have us believe. A few days later I visited this same gentleman's clinic, which is a large one, and in two hours I found six pairs of old, sequestrated Fallopian tubes, full of pus, carried around by six of the most careworn and miserable-looking women imaginable. They had their vaults frescoed with Churchill and were directed to return for another decoration the following week.

Conservatism is a grand principle, but, unfortunately, in the hands of skilful men the application of this rule is only too frequently responsible for destructive or complicative delays, if I must use such a term. Conservatism is a prophylactic if early and intelligently carried out or applied. It is equally injurious if used with this same idea in view, in cases where time and experience have demonstrated its futility.

Occasionally an article appears in some of the many valuable medical journals of our country, entitled A Plea for Conservatism, etc., etc. That these articles are *well worded* by conscientious practitioners, in most instances, no one will doubt, and to one unaccustomed to seeing the true pathology from a practical standpoint, they carry with them weighty evidence that, with rare exceptions, all surgical procedures for the relief or cure of same are unnecessary and unwarrantable ; but to him who has handled these cases surgically, and understands their progress, the position of the so-called conservative is not well taken. Many are attempting to do this class of surgery (pelvic) who have not the anatomical or pathological knowledge or practical experience to enable them to make a diagnosis between an operable or non-operable case. To this class, sermons on qualifications and attainments should take the precedence of lectures on operative conservatism.

Good missionary work is being done in this field by educating the general practitioner and the specialist that they should go hand in hand in their work. By friendly discussions and exchange of views on topics of vital import to both, the patients reap the benefit of the combined council.

We have men of renown in this country as operators and authors, whose utterances along the line of so-called conservatism are producing much mischief and causing many deaths by the adoption of these false doctrines by lesser lights. After seeing much of the work of some of these men, I am surprised to see some of their ideas in print so foreign to the practice actually pursued by them in their work.

One is almost tempted to doubt the sincerity of some of their utterances.

We should advise against the removal of sound organs, but, at the same time, endeavor to impress upon our associates the necessity for early surgical work where experience with like cases has demonstrated the futility of any other course.

Operations for the removal of diseased appendages are not followed by the same amount of reflex disturbance as are those cases where sound organs are removed to cure (?) a clavus hystericus or a globus hystericus, etc., etc.—an unwarrantable procedure. Most women with suppurating, diseased tubes and ovaries are unsexed by the pathology, and I have had women with these diseases at an age too early for a normal menopause, to present all the climacteric phenomena. In many cases it is not a question of, "Will this woman have an exaggeration of the menopause phenomena if we operate?" so much as, "Will she be free from pain after the operation, and can she recover without the aid of surgery?" These questions have been answered many times, both by non-interference in the cases, with failures and by demonstrations at the operating-table,—the successes. Women whose appendages are diseased to such an extent as to require removal are sick women, and are necessarily in that low vital state that where there is an inherent tendency to insanity or neurasthenia, the surgical procedure for the removal of the diseased structures may precipitate the mental phenomena to an exaggerated and disagreeable degree.

If we are not to remove life-endangering and comfort-breaking pathological processes when found, pray tell us what are we to do? Fold our hands and let nature (pathology) take its course? Or do worse, visit these cases with poultices, Churchill and hypodermics? We can illy afford, as true surgeons, to cater to the plea that the poor sufferer will be robbed of her womanly traits; that she will be despondent, lose her sexual desires, suffer from flushes and flatulence, and that her husband (too often responsible for the wife's sufferings) will become dissatisfied with the post-operative condition of his wife. I have never seen a woman suffering with diseased appendages, where the disease was of sufficient severity to require the removal of the organs, who was not a sexually useless and despondent wife, and a sick woman as well. Sound organs should not be removed. Any condition that may be induced by the surgical procedure may likewise be induced by a continuance of the disease requiring the surgery.

I believe the sexual system is located elsewhere than in the tubes and ovaries. The nymphomaniac manifests not only an increased sexual desire, but a loss of self-control, often with hallucinations and delusions, the whole cycle having its origin in the cerebrum.

Radicalism is a dangerous expression to use; equally harmful is the term conservatism applied to a disease the tendency of which is to destroy function, make life miserable or produce death if too long a delay of a proper procedure for its cure is permitted.

Many cases early in the history of the malady are prematurely cured by intelligent and skilled surgery. The same case, if allowed to run an uninterrupted course for any length of time, may assume proportions or characters of such magnitude or danger that to attempt to relieve the same would not only be fraught with danger from the operation, but the crippled surrounding organs would preclude the possibility of a complete restoration to health.

These are cases that bring disappointment to the patients and friends, as well as to the sanguine physician and surgeon, run up the mortality and give surgery a "black eye."

It is the early removal of diseased structures, the history of which is to continue a downward course, that gives us a *nil* mortality, the patient renewed health and the family physician increased confidence in the justifiability of the operative procedure.

Early operations are not only curative measures, but prophylactic methods as well. It is not only the diseased and worse than useless organs and structures the true abdominal surgeon is removing, but he is liberating sound imprisoned organs, the function of which is essential to life. This is not only life-saving work, but a comfort-giving procedure. No *true surgeon* is clamoring for a thousand or ten thousand laparatomies, but is ever pleading for timely needed work—is ever pleading for the recognition of the necessity for quick surgery in cases where surgery at some time in the history of the malady becomes necessary.

No one at this time would think of advising a woman to wait until the growth was so large or her physical condition such that she could not walk a mile, before having the tumor removed, as was done a few years ago by a great writer. This would be dangerous conservatism.

It is as absurd to call the removal of a sequestrated Fallopian tube and ovary a mutilation as it is to call an amputation of a hopelessly injured leg a mutilation. These operations are performed to fulfil strictly surgical indications—saving life and relieving suffering. It is a daily occurrence to see reported that a case diagnosticated as appendicitis has recovered. That this is true no one will doubt. Many cases reported as recovered have since died from recurrence of the disease, and many an appendix, supposed by the medical attendant to be dangling healthfully in the peritoneum of his patient, is saturated with Muhler's fluid or alcohol, in a specimen jar, while the patient has long since died from a perforation, or has been saved by good, timely surgery. No operation in surgery has a higher mortality than that of the delayed operations for the removal of a diseased vermiform appendix, but if done early, the procedure has an almost *nil* mortality. A late writer reports twelve cases treated on the expectant plan with two deaths (16 per cent. mortality) and two cases with recurrent attacks (16 per cent. of recurrences). The writer says of the two who died that they were almost in *articulo mortis* when first seen by him. He does not tell us whether he put on a fresh poultice and hid from view the big abscess bulging in the right iliac fossa, or gave the patient an extra dose of opium to relieve the pain of the patient and obtund the senses of the doctor. These cases should have been seen by a good surgeon, who would have stopped all opiates and let out the pus, applying the same surgical principles to this locality as to other parts of the body. "When you find pus, let it out." Appendicitis is a surgical disease, and the surgeon should see the case with the physician as soon as the diagnosis of appendicitis is made. Let the physician and the surgeon watch the case together.

The modern application of the principles of surgery as applied to diseased Fallopian tubes is the same that has been practised for ages as applied to the surface of the body. A felon is lanced—the earlier the better. The good surgeon does not advise delay and, while waiting, paint the finger with Churchill or apply hot irrigation. As soon as evidences are present that pus is forming, he lets it out.

How thankful the abdominal surgeon would be could he lift up a pus-filled Fallopian tube without entering the peritoneum, "rip it up the back" (as suggested by a late writer), scrape it out, destroy all the remaining epithelium, and by redressing it restore its caliber so that the spermatozoa may throng its canal and pregnancy take place, just as though this culture tube had not been a hotbed for gonococci and other pathogenic bacteria for months previous.

All cases of salpingitis are not operable cases, but the majority of cases seen by the specialist are old purulent cases with tubes filled with pus and caseous débris strictured in one or more places, uterine and abdominal ostii closed. Here it is conservatism to remove these dangerous sequestræ.

Hardly a week passes without seeing one or more cases of far-advanced cancer of the cervix uteri extending into the broad ligaments, bladder or rectum. These are inoperable cases when so far advanced. Again, I see cases where a diagnosis of a cauliflower growth has been made, which proves to be a badly lacerated cervix, with the resulting local mischief attending an unhealed tear in this locality. Surgical diseases should be attended by the surgeon in conjunction with the regular medical attendant. The time to operate, if necessary at all, could then be arrived at *mutually* and *timely*.

When the masses are educated in every sense, when gonorrhea is stamped from the face of the globe, when the perniciousness of criminally induced abortions and the dangers of the indiscriminate use of the sound are understood, then there will be less necessity for the practitioner to hurl at the specialist his "bucket or barrel of ovaries" (?); then the specialist will cease his cry against needless tinkering and dangerous procrastination; then our women will have their babies at full term, in the good old way. Until this goal is reached, the use of the knife will be found necessary in properly selected cases to relieve the suffering and save the lives of these unfortunate women with diseases the result of the above mentioned causes.

